# Supplementary material for: Better coverage, better outcomes? Mapping mobile network data to official statistics using satellite imagery and radio propagation modelling
Source: PLoS One. 2020 Nov 9;15(11):e0241981. doi: 10.1371/journal.pone.0241981 (PMC7652289; doi:10.1371/journal.pone.0241981)
Supplement: S1 Appendix — Results from the cross-checks of the application and instructions for replicating the findings of this study. (PDF) [file pone.0241981.s001.pdf]

S1 Appendix - Better coverage, better outcomes? Mapping mobile network data to official statistics using satellite imagery and radio propagation modelling

Till Koebe[1*]

1 Department of Economics, Freie Universität, Berlin, Germany

* till.koebe@fu-berlin.de

This document provides results from the cross-checks of the application found in the paper "Better coverage, better outcomes? Mapping mobile network data to official statistics using satellite imagery and radio propagation modelling". In addition, the document provides instructions for replicating the results of this study.

Cross-checks of application results

Rural-urban performance differences

As stated in the application of the paper, urban communes do not perform significantly better than rural ones as suggested by the simulation results. A possible reason could be that the estimation of urban areas is less robust as there are less urban communes than rural ones in Senegal. Tables 1 and 2 show the results for in-sample and out-of-sample predictions by commune status, respectively.

Table 1. In-sample area-level correlation of estimated and true unemployment rate & sample size.

Mapping	ρ	n	ρ_{Rural}	n_{Rural}	ρ_{Urban}	n_{Urban}
Point	0.765	191	0.745	176	0.789	16
Voronoi	0.778	196	0.759	180	0.786	16
Aug. Voronoi	0.780	195	0.762	179	0.777	16
Simple HATA (BSA)	0.770	194	0.750	178	0.783	16
Simple HATA (IDW)	0.771	196	0.751	180	0.784	16

Table 2. Out-of-sample area-level correlation of estimated and true unemployment rate & sample size.

Mapping	ρ	n	ρ_{Rural}	n_{Rural}	ρ_{Urban}	n_{Urban}
Point	0.320	210	0.275	180	0.308	30
Voronoi	0.313	235	0.283	205	0.297	30
Aug. Voronoi	0.280	233	0.246	203	0.211	30
Simple HATA (BSA)	0.269	232	0.238	202	0.141	30
Simple HATA (IDW)	0.308	234	0.285	204	0.225	30

Classification error in the settlement data

Even though GUF data is supposed to have a true positive rate of 85 % on average, with 68 % at lowest and 98 % at heighest, two Senegalese communes host no settlements. This hints at the presence of classification error in the settlement data that may lead to efficiency losses in the estimation process. To investigate it further, I use an alternative source of settlement information. While GUF data is generated from satellite imagery only (180.000 single TerraSAR-X/TanDEM-X image products) at 0.4 arcseconds, WPG data is based on Random Forest-based dasymetric mapping approach using a wide variety of input data including land-cover information from MERIS imagery, night-time lights, distance information to various thematic land-cover classes etc at approx. 3 arcseconds.

Table 3. Best performing approach for *unemployment rate* by round across rounds (in %).

Mapping	Adj. R^2	Bias		RMSE		Avg. # of predictors
	in	in	out	in	out	
Point	4.6	12.4	15.2	9.0	40.8	4.2
Voronoi	6.2	13.0	10.0	12.2	15.4	5.0
Aug. Voronoi (GUF)	18.2	16.4	10.4	17.6	2.2	6.5
HATA (GUF, BSA)	17.2	16.8	10.2	9.4	4.6	6.4
HATA (GUF, IDW)	18.6	9.8	15.2	6.0	6.4	6.2
Aug. Voronoi (WPG)	12.0	11.4	13.2	13.0	7.2	6.0
HATA (WPG, BSA)	12.8	14.0	14.0	17.2	5.4	5.9
HATA (WPG, IDW)	10.4	6.2	11.8	15.6	18.0	5.0

Additional outcomes of interest

The following two tables provide results for two additional outcomes of interest, in this case the literacy rate and the population count.

Table 4. Best performing approach for *literacy rate* by round across rounds (in %).

Mapping	Adj. R^2	Bias		RMSE		Avg. # of predictors
	in	in	out	in	out	
Point	59.2	14.6	93.8	70.4	77.4	5.6
Voronoi	1.0	13.0	0.6	0.8	0.2	5.1
Aug. Voronoi (GUF)	4.2	10.2	1.0	10.6	19.4	5.3
HATA (GUF, BSA)	6.8	7.4	0.8	1.6	0.4	5.5
HATA (GUF, IDW)	1.0	5.4	1.8	1.8	0.8	4.9
Aug. Voronoi (WPG)	14.6	10.4	1.8	3.6	0.8	5.6
HATA (WPG, BSA)	8.2	7.2	0.2	10.8	0.8	5.5
HATA (WPG, IDW)	5.0	41.2	0.0	0.4	0.2	5.5

Table 5. Best performing approach for *population count* by round across rounds (in %).

Mapping	Adj. R^2	Bias		RMSE		Avg. # of predictors
	in	in	out	in	out	
Point	23.4	17.8	5.4	14.6	0.0	6.1
Voronoi	0.0	0.2	85.4	0.2	3.4	3.2
Aug. Voronoi (GUF)	0.0	27.8	0.0	2.0	0.0	3.0
HATA (GUF, BSA)	0.2	10.2	0.0	39.4	0.0	3.8
HATA (GUF, IDW)	73.8	36.8	0.0	26.8	0.0	9.0
Aug. Voronoi (WPG)	1.2	4.0	0.0	15.6	5.2	5.3
HATA (WPG, BSA)	0.4	3.2	0.2	1.4	11.2	4.8
HATA (WPG, IDW)	1.0	0.0	9.0	0.0	80.2	4.2

Replicating the simulation

The complete code for replicating the simulation can be found as 'S1 File' in the supporting information of this paper as well as in the following GitHub repository:
`https://github.com/tilluz/geomatching_open`. No additional files are required.

Replicating the application

The complete code and necessary data for replicating the application can be found as 'S2 File' in the supporting information of this paper as well as in the following GitHub repository:
`https://github.com/tilluz/geomatching_open`. The replication results may slightly differ from the results presented in this study as it cannot be ensured that the 10%-sample of the census data provided by the statistical office of Senegal are identical to the one used in this study. Following data sources not available in the repository are necessary in order to run the code:

- *spss_car_individus_10eme_dr.sav*: The 10% sample of the population part of the RGPHAE 2013. Access can be requested via the microdata portal of the statistical office of Senegal (ANSD):

http://anads.ansd.sn/index.php/catalog/51/

- *SITE_ARR_LONLAT_EXACT.csv*: This file contains the exact tower locations of SONATEL in 2013. Access can be requested as stated in the data availability statement of this study. To facilitate replication, a file with slightly randomized antenna locations is provided (*SITE_ARR_LONLAT.csv*). Keep in mind this may affect the final outcomes. The exact locations have to be requested as stated in the data availability statement.

- *sen_ppp_2013.tif*: This file contains the population density estimates of Senegal for the year 2013. The data can be downloaded at the WorldPop website:
  https://www.worldpop.org/doi/10.5258/SOTON/WP00645

- *senegal.tif*: This file contains the GUF data for Senegal at 0.4 arcseconds. Access can be requested for scientific, non-commerical purposes via the website of the German Aerospace Center DLR:
  https://www.dlr.de/eoc/en/PortalData/60/Resources/dokumente/guf/DLR-GUF_LicenseAgreement-and-OrderForm.pdf

The application is written in Python and R. The file and folder names indicate the required order of execution. Files 04 - 07 may not be run unless access to individual-level CDRs is available. It may be necessary to align directory paths to make the code run properly. Please ensure that more than 20GB RAM is available for this analysis. If needed, please contact the corresponding author for support.
