# Supplementary material for: Better coverage, better outcomes? Mapping mobile network data to official statistics using satellite imagery and radio propagation modelling
Source: PLoS One. 2020 Nov 9;15(11):e0241981. doi: 10.1371/journal.pone.0241981 (PMC7652289; doi:10.1371/journal.pone.0241981)
Supplement: Attachment — Submitted filename: Response to Reviewers.pdf [file pone.0241981.s004.pdf]

Response to Reviewers - Better coverage, better outcomes? Mapping mobile network data to official statistics using satellite imagery and radio propagation modelling

Till Koebe[1*]

1 Department of Economics, Freie Universität, Berlin, Germany

* till.koebe@fu-berlin.de

I am grateful to the academic editor and the two reviewers for their constructive and excellent comments. These have been very helpful for improving and preparing the revised version of this paper. I have done my best to respond to all comments. The following points show how I addressed each comment.

Replies to the Academic Editor

- Please ensure that your manuscript meets PLOS ONE's style requirements, including those for file naming.
  Reply: In order to meet the style requirements, I added 'Corresponding Author' including the initials to the author information, I made figure captions bold and I aligned the panel labels in the text according to the figures.

- We note that Figures 1, 8, 9 in your submission contain map images which may be copyrighted. All PLOS content is published under the Creative Commons Attribution License (CC BY 4.0), which means that the manuscript, images, and Supporting Information files will be freely available online, and any third party is permitted to access, download, copy, distribute, and use these materials in any way, even commercially, with proper attribution. For these reasons, we cannot publish previously copyrighted maps or satellite images created using proprietary data, such as Google software (Google Maps, Street View, and Earth).[...] We require you to either (1) present written permission from the copyright holder to publish these figures specifically under the CC BY 4.0 license, or (2) remove the figures from your submission. [...]

Reply: Thank you for this important comment. As the built-settlement information from the Global Urban Footprint project used in this study with a resolution of 0.4 arcseconds (∼12 x 12 meters per pixel) are available for scientific, non-commercial use only, I replaced Figures 1 and 8 with built-settlement information under CC BY 4.0 licence from WorldPop at 3 arcseconds (∼100 x 100 meters per pixel) for illustrative purposes. Further, Figure 9 is intended to give initial indication how well aligned the coverage map used in the application of this paper is with the official coverage map provided by the mobile network operator. However, the official coverage map is not available under a CC BY 4.0 licence. Consequently, I removed Figure 9 accordingly. However, this does not affect the substance of the application study.

- We note that you have indicated that data from this study are available upon request. PLOS only allows data to be available upon request if there are legal or ethical restrictions on sharing data publicly.
  - If there are ethical or legal restrictions on sharing a de-identified data set, please explain them in detail (e.g., data contain potentially sensitive information, data are owned by a third-party organization, etc.) and who has imposed them (e.g., an ethics committee). Please also provide contact information for a data access committee, ethics committee, or other institutional body to which data requests may be sent.

    Reply: The mobile phone data at the antenna- and commune-level aggregated to the year 2013 as well as instructions for replicating the study results have been added as part of the supporting information. In order to access record-level data, one would need to contact Sonatel directly and present the research project that would require the data (contact: Mr El Hadji Birahim Gueye, Direction des Systèmes d'information Sonatel, ebgueye@orange-sonatel.com or post mail: Orange-Sonatel, 46 Boulevard de la République, BP 69 Dakar, Senegal).
  - If there are no restrictions, please upload the minimal anonymized data set necessary to replicate your study findings as either Supporting Information files or to a stable, public repository and provide us with the relevant URLs, DOIs, or accession numbers.

    Reply: Please see my reply above.

Replies to Reviewer #1

- As a general comment, I believe the authors have understated one of the main advances the paper offers, which is to allow for the idea that the statistical units at which poverty is measured may be best represented by more than one tower location. This idea should be better expressed in the introduction.

  Reply: Thank you for this excellent comment. While the mentioned advancement is stated quite clearly in the 'Contributions' section, the corresponding problem statement has not been spelled out properly indeed. Consequently, I added the following sentence and an example to the introduction to make this advancement more clear: *[Secondly,]even though the concept of 'home-locating' subscribers to specific BTS offers a network-based alternative to the statistical concept of 'usual place of residence', it is not reflected within cells. [...] For example, an uninhabited lake would receive the same importance in the creation of area-level mobile phone metadata aggregates as an equally sized built-up area.*

- Both settlement weighting to derive augmented Voronoi polygons and radio-propagation-based modelling aim to address a core problem with how population density interacts with tower densities. While the results in the present study do not show significant improvements in predictive power, I feel that it could be valuable to further explore the relative contribution of settlement weighting and radio-propagation modelling in the context of population density and tower distribution. For example, in the simulation studies, the predictive power of the model was higher in urban versus Rural areas. It would be of interest to see how this difference played out in Senegal.

  Reply: I added the following sentence to the simulation study to make this point on the relative contributions more clear: *Performance differences between voronoi tessellation versus the augmented voronoi tessellation and the augmented voronoi tessellation versus the HATA (BSA) approach showcase the relative contribution of settlement weighting and radio-propagation modelling, respectively.* Further, I added a paragraph including both a table (Table 8) similar to Table 5 and a scatterplot (Fig. 10) showing the true versus the estimated unemployment rate by commune status (urban vs. rural) to the application part to better discuss and illustrate the differences in performance disaggregated by commune status (urban / rural).

- In the conclusions, the author suggests that model misspecification could be the reason for a lack of significant model improvement using propogation models. A further potential interpretation – and possible contribtion of the study is that it hints at a lower limit to the scale at which spatial

heterogeneity in poverty rates can be discerned using CDR data, at least in the context of poverty data aggregated within statistical units.

Reply: This is an important and interesting line of thought. As an adequate investigation of this question goes beyond the scope of the current paper, I adapted the conclusion accordingly to account for it as further research: *As the lower bound of spatial heterogeneity captured is given by the number of unique areas resulting from intersecting coverage areas and statistical areas, studies~~, e.g.~~such as [4] have shown that moving from an BTS-oriented to a cell-oriented analysis could greatly affect analysis, especially via potential increases in sample size. However, it needs further investigation how refined mapping schemes can add further value, particularly in the presence of measurement uncertainty, to supervised learning setups in cell-level analysis.*

- Please be consistent with capitalisation of 'Voronoi'

  Reply: Thanks for the hint. Similar to 'Point-to-Polygon', I capitalize 'voronoi' only in figures and tables as 'Aug. Voronoi' being the abbreviated form of 'augmented voronoi tessellation' and therefore a standalone term in my view. For consistency and style reasons, I would leave it that way.

- Line 196: Please define HATA the first time you use it in text. Or does HATA refer to the author in citation 53?

  Reply: To make this more clear, I added the following by-sentence to the first mentioning of HATA: *[...], named after Masaharu Hata, the author of the 1980 landmark study on the "Empirical Formula for Propagation Loss in Land Mobile Radio Services"*

- Line 332: Please define BSA as Best server area here.

  Reply: Thanks for the suggestion. I added the abbreviation accordingly.

- Line 350: please define IDW here as inverse distance weights

  Reply: Same here.

- Line 596: I don't see any differentiation between urban and rural predictions in the Senagal case. Can these be provided for interest?

  Reply: Yes, I added and discussed them, accordingly. For details, please see the reply above.

- Figure 2: please be consistent in your labelling of the different scenarios so that 2e and 2f are labelled BSA and IDW in line with other text and tables.

Reply: Thanks again for the hint. I added the abbreviation (BSA) and (IDW) accordingly to be consistent with other tables and figures of this study.

Replies to Reviewer #2

- My most pressing concern is that the application in Senegal tests only one outcome measure – unemployment – while making broad statements about the usefulness of the proposed methodology. While unemployment is a useful measure to estimate using small area estimation (SAE) methods, it's hardly the only one. I would recommend including additional outcome variables – such as poverty, average income, population, crop output, etc. – to test the comparison across methods. The author mentions she uses literacy rate as an outcome, but does not present model diagnostics. I would rethink the tables in this section and present across a range of outcome measures before making such strong statements in the abstract and intro regarding the usefulness of this proposed method.

  Reply: This comment is very useful. I added the literacy rate and the population count as additional outcomes including corresponding model diagnostics to the supporting information of this submission alongside other cross-checks run in the results section of the application. The additional outcomes underline the main takeaway of the paper - that better coverage approximations do not necessarily lead to better outcome predictions.

- The small area estimation model used in Senegal to estimate unemployment could use some adjustment. I advise against the use of Bayesian Information Criterion as a method of model selection in the application in Senegal. Incidentally, this could result in the lack of improvement in out of sample power. BIC is not robust to overfitting, and out of sample methods such as Ridge or Lasso should be used (see Blumenstock et al., 2015). I also would like to see more diagnostics on the SAE model, specifically the number of variables that could be selected, as well as model fit.

  Reply: Thank you for the suggestion. I re-ran the application with LASSO instead of BIC for model selection and updated the outputs accordingly. While the number of average predictors has halved, there is no clear winner concerning the indicators of model fit (cf. Table 6). Further, I added the total number of available covariates to the text (in fact, 72 covariates are available before model selection).

- On page 4 you state you only use a 10% sample of the RGPHAE 2013 to derive commune-level

unemployment. Why not use a 100% sample? The variance of the estimate will decrease with the sample size.

Reply: I fully agree, however, the statistics office of Senegal (ANSD) solely provides 10% samples of their census data via their microdata portal.

- I would appreciate more description of what radio propagation models are in the introduction for the sake of development economists who may not be familiar with this.

  Reply: I added the following sentence to the introduction that clarifies the link between the topic of coverage area estimation and radio propagation: *Third, as mobile stations (MS, generally defined as a combination of device and SIM card) and antennas communicate via modulated radio signals whose propagation paths depend on a range of factors such as the weather, coverage areas are stochastic by nature.* Further details on radio propagation modelling are then provided in the subsection on 'Radio propagation modelling'.

- Similarly, could you describe the intuition of equation (2) again for the uninitiated.

  Reply: I added the following sentence to provide some intuitive explanation of equation 2: *Intuitively, Eq. 2 thus states that the signal strength observed on a MS solely depends on the output power of the connected antenna and the loss in signal strength that occurs along the way between antenna and MS.*

- On page 9 line 214: is this path loss big or small? Can you please comment on the magnitude?

  Reply: Thank you for the question. While the answer to that question is actually given right after, I made it more clear in the text now that the path loss in question is further used to calculate the received signal strength by explicitly recapping Eq. 2 ($P_{rx} = P_{tx} - L_p^{EH} \approx -75dBm$).

- More description of figure 2 in the text would be helpful.

  Reply: Thank you for the suggestion. The three subsections following Fig. 2, i.e. 'Point-to-polygon allocation', 'Voronoi tessellation' and 'Augmented voronoi tessellation' provide detailed explanation of this figure. To provide additional guidance to the reader, I added the following sentence right before Fig 2: *Further details on Figs 2b - 2f are provided in the following subsections.*

- In general, the labels of the figures were lacking and I'd appreciate more description in their titles or figure notes.

Reply: Thank you for the remark. I aligned the capitalization of the panel labels to correspond to the figures. Also, I added further descriptions in the captions of Figs 1, 2, 3, 7, 8 and 9.
